# A risk prediction algorithm for ovarian cancer incorporating *BRCA1, BRCA2*, common alleles and other familial effects

**DOI:** 10.1136/jmedgenet-2015-103077

**Published:** 2015-05-29

**Authors:** Sarah Jervis, Honglin Song, Andrew Lee, Ed Dicks, Patricia Harrington, Caroline Baynes, Ranjit Manchanda, Douglas F Easton, Ian Jacobs, Paul P D Pharoah, Antonis C Antoniou

**Affiliations:** 1 Department of Public and Primary Care, Centre for Cancer Genetic Epidemiology, University of Cambridge, Cambridge, UK; 2 Department of Oncology, Centre for Cancer Genetic Epidemiology, University of Cambridge, Cambridge, UK; 3 Institute for Women's Health, University College London, London, UK; 4 Department of Gynaecological Oncology, St Bartholomew's Hospital; 5 Faculty of Medical & Human Sciences, Institute of Human Development, The University of Manchester and Manchester Academic Health Science Centre

**Keywords:** Genetic epidemiology, Ovarian Cancer, Risk prediction, Genome-wide, Genetic screening/counselling

## Abstract

**Background:**

Although *BRCA1* and *BRCA2* mutations account for only ∼27% of the familial aggregation of ovarian cancer (OvC), no OvC risk prediction model currently exists that considers the effects of *BRCA1, BRCA2* and other familial factors. Therefore, a currently unresolved problem in clinical genetics is how to counsel women with family history of OvC but no identifiable *BRCA1/2* mutations.

**Methods:**

We used data from 1548 patients with OvC and their relatives from a population-based study, with known *BRCA1/2* mutation status, to investigate OvC genetic susceptibility models, using segregation analysis methods.

**Results:**

The most parsimonious model included the effects of *BRCA1/2* mutations, and the residual familial aggregation was accounted for by a polygenic component (SD 1.43, 95% CI 1.10 to 1.86), reflecting the multiplicative effects of a large number of genes with small contributions to the familial risk. We estimated that 1 in 630 individuals carries a *BRCA1* mutation and 1 in 195 carries a *BRCA2* mutation. We extended this model to incorporate the explicit effects of 17 common alleles that are associated with OvC risk. Based on our models, assuming all of the susceptibility genes could be identified we estimate that the half of the female population at highest genetic risk will account for 92% of all OvCs.

**Conclusions:**

The resulting model can be used to obtain the risk of developing OvC on the basis of *BRCA1/2*, explicit family history and common alleles. This is the first model that accounts for all OvC familial aggregation and would be useful in the OvC genetic counselling process.

## Introduction

Ovarian cancer (OvC) is the third most common gynaecological cancer (http://www.cancerresearchuk.org/cancer-info/cancerstats/). It is well-established that OvC has a significant genetic component, with the risk to first-degree relatives of patients with OvC estimated to be approximately three times greater than the risk to women in the general population.^1^ ^2^ High-penetrance mutations in *BRCA1* and *BRCA2* account for ∼27% of these familial cancers^1^ and another 10% are accounted for by rare variants in the MMR genes, *RAD51C, RAD51D* and *BRIP1* (http://www.nature.com/icogs/primer/common-variation-and-heritability-estimates-for-breast-ovarian-and-prostate-cancers/).

Risk models that incorporate both *BRCA1* and *BRCA2* mutations and other sources of variation are required to provide accurate estimates of mutation carrier probabilities and cancer risk for use in genetic counselling. Existing risk-prediction models for familial OvC such as Breast and Ovarian Analysis of Disease Incidence and Carrrier Estimation Algorithm (BOADICEA) or BRCAPRO^3^ ^4^ assume that all familial aggregation to OvC is due to *BRCA1* and *BRCA2* mutations but this does not reflect our understanding of OvC genetic susceptibility. As a consequence, these models may underestimate OvC risks in women without mutations in these genes. Therefore, how to counsel women with family history of OvC but without *BRCA1* or *BRCA2* mutations has remained a major unresolved question in clinical cancer genetics.

We have used data from a large, population-based series of cases diagnosed with OvC, the Studies of Epidemiology and Risk factors in Cancer Heredity (SEARCH), and segregation analysis methods to develop genetic models for OvC that incorporate the effects of *BRCA1* and *BRCA2* mutations and model the residual familial aggregation to OvC. The explicit effects of 17 common OvC susceptibility alleles, identified through genome-wide association studies (GWAS), were then incorporated into the algorithm. We finally considered the implications of our risk prediction model for OvC risk stratification in the general population and its use in OvC prevention.

## Materials and methods

### Study population

We used data on 1548 OvC cases (probands) recruited between 1999 and 2010, along with information on their first-degree and second-degree relatives ascertained through an epidemiological questionnaire. The probands were drawn from SEARCH, a large population-based study with cases ascertained through the Eastern Cancer Registration and Information Centre.^1^ ^5^ Half-sibling status and relative type to the proband, age at cancer diagnosis, cancer site, vital status, status age (the age at death if deceased, the current age if alive) and year of birth were recorded for all probands and relatives.

### *BRCA1* and *BRCA2* mutation screening

SEARCH OvC probands were screened for *BRCA1* and *BRCA2* mutations as part of a separate project to evaluate the contribution of rare, high-risk and moderate-risk variants to overall OvC risk in the general population.^6^ Briefly, this involved targeted sequence library preparation using multiplexed 48.48 Fluidigm access arrays and sequencing on an Illumina HiScan. *BRCA1* and *BRCA2* mutation status information was available on all 1548 probands. The following alterations were considered pathogenic: protein-truncating insertion/deletion variants, nonsense mutations, consensus splice-site variants and missense variants with reported damaging effect on protein function. For the purpose of our analysis, *BRCA1* and *BRCA2* mutation status were both recorded simply as mutation-positive or negative, with no distinction between different mutation types by location or functional effect.

### Statistical analysis

#### Segregation analysis of OvC

Complex segregation analysis was used to fit genetic models to the occurrence of OvC in families, incorporating the explicit effects of *BRCA1* and *BRCA2* mutations on OvC risk. Female family members were followed from birth until the first of OvC diagnosis age, age at questionnaire, death age or age 80. We also considered breast cancer occurrence, but individuals were continued to be followed up for OvC after a breast cancer diagnosis in the analysis. Data on risk-reducing surgeries were not available in relatives of probands, and we were therefore unable to censor at these events. However, since this is a population-based study in which women with OvC diagnosis were recruited soon after diagnosis, and participants were not aware of their mutation status at the time of recruitment, we do not expect a high prevalence of risk-reducing surgeries at the time of pedigree collection.

To incorporate the effects of *BRCA1* and *BRCA2* mutations and to take account of changes in cancer incidences over time, the OvC incidence for a female i was assumed to depend on the underlying genetic effects through a model of the form
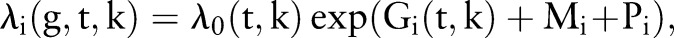
where 
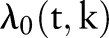
 is the baseline incidence for individuals born in birth cohort k and 
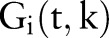
 is the logarithm of the relative risk associated with *BRCA1/BRCA2* mutation status g*,* for age t and cohort k*;* M_i_ is the logarithm of the relative risk associated with a third hypothetical major gene and P_i_ is the polygenic component. P_i_ is assumed to have a normal distribution with variance σ^2^ and mean zero and is approximated by the hypergeometric distribution to make it amenable to ‘peeling’.^7^ ^8^ Eight sets of birth cohort and calendar-period-specific incidences for OvC in the general population were derived on the basis of incidences for England and Wales as described previously for the BOADICEA model.^9^ The eight cohorts included individuals born pre-1920 then in 10-year intervals up to post 1970. As the number of *BRCA1* and *BRCA2* mutation carriers in the SEARCH dataset was too small to obtain reliable cancer risk estimates for mutation carriers, we also used external estimates of the OvC and breast cancer relative risks for *BRCA1* and *BRCA2* carriers relative to population incidences, based on some of the largest studies available.^10^ Hence, the average *BRCA1* and *BRCA2* OvC and breast cancer incidences over all possible genetic effects in the model were fixed. The cohort-specific baseline incidences for *BRCA1* and *BRCA2* carriers were obtained for each cohort separately by constraining the average incidences over all possible genetic effects to agree with the external estimates.^8^ Similarly, the baseline incidences for non-mutation carriers were obtained by constraining the incidences over the *BRCA1, BRCA2,* other major gene and polygenic effects to agree with the population incidences (see online supplementary material methods).

Since *BRCA1* and *BRCA2* mutations are also associated with increased breast cancer risks,^10^ ^11^ we incorporated the effect of these mutations on breast cancer incidence. We assumed a similar model for the breast cancer incidence; however, the breast cancer incidence was assumed to depend on only the effects of *BRCA1* and *BRCA2* mutations.

In our analyses, we considered models with just the *BRCA1* and *BRCA2* effects, and models that additionally included a dominant, recessive or co-dominant hypothetical major gene and/or a polygenic component.

All the families used in the analysis consisted of women ascertained on the basis of OvC. Thus, to adjust for ascertainment bias,^12–14^ we employed an ascertainment assumption-free approach in which the likelihood of each family's joint phenotype was modelled as 
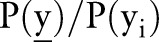
, where 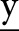 is the vector of all the family phenotypes including all phenotypic and genotypic information on the proband and y_i_ is the phenotype of the proband. A sensitivity parameter was introduced, giving the probability of detecting a mutation if one existed, to take account of the fact that mutation screening methods used cannot detect large rearrangements in *BRCA1* and *BRCA2.*^6^ A fixed value of 0.9 for both *BRCA1* and *BRCA2* was used in all models, but additional sensitivity analyses were performed.

Maximum-likelihood estimates of the gene frequencies, polygenic standard deviation and the log relative risk for the hypothetical major gene were calculated using pedigree analysis software MENDEL.^15^ SEs for each parameter were obtained from the observed information matrix and were used to calculate 95% CIs. To assess goodness of fit, all of the models with a polygenic or major gene component were compared with the baseline model with just *BRCA1* and *BRCA2* effects using likelihood ratio tests (LRTs). Further LRTs were used to test for differences between the fit of nested models and the Akaike information criterion (AIC) equal to −2(log-likelihood – no. of parameters) was used to compare non-nested models.

#### OvC risk, mutation frequency and carrier numbers prediction

We used each of the models fitted to predict *BRCA1* and *BRCA2* mutation carrier frequencies and the risk of developing OvC in the future using the methods previously described in ref [11]. The predictions were used to compare the fit of the models as part of an internal validation. Although goodness-of-fit tests are not valid using the data generating dataset, we calculated χ^2^ goodness-of-fit tests that compared the observed and expected number of mutations and used these as an indicator of the model fit to the data. The expected number of mutation carriers was computed as the sum of the predicted *BRCA1* and *BRCA2* carrier probabilities across all SEARCH families.

We used the most parsimonious model to estimate risk of developing OvC for a 50-year-old woman to demonstrate the possible clinical implications for different scenarios of BRCA1 and BRCA2 carrier status and extent of family history. The results were compared with the corresponding predictions from the current BOADICEA model.

#### Incorporating SNPs into the risk prediction algorithm

We extended the most parsimonious model to also incorporate the explicit effects of the known common OvC susceptibility alleles following the methodology already published in the context of prostate cancer.^16^ The residual familial aggregation of OvC was accounted for in this model by a polygenic component reflecting the additive effects of a large number of genetic variants. The polygenic component P_i_ for each individual was divided into two parts for this purpose: a known-variant polygenic component P_k,i_ reflecting the polygenic risk score (PRS) due to 17 SNPs known to be associated with OvC^17^ and an unknown residual polygenic component P_U,i_. The two components were assumed to be independent and normally distributed with mean 0 and variance 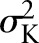 and 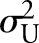, respectively (see details in online supplementary material; methods). 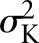 was calculated using previously described methods,^16^ based on the known allele frequencies and per-allele OR estimates. 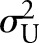 is then obtained as the difference between the total polygenic variance and variance of the PRS.

#### Distribution of OvC risk and implications for OvC prevention

The OvC risk associated with any individual common genetic variant is very small compared with rare variants like *BRCA1.* However, as there are thought to be many as yet undiscovered common variants and their effects are assumed to be additive on the logarithmic scale a woman with a high polygenic load is likely to have a greatly increased risk of OvC compared with someone with a low polygenic load. Being able to distinguish between high-risk and low-risk individuals in the population could be a valuable tool in clinical practice. Therefore, we considered the potential for risk prediction based both on known common variants and the total hypothesised polygenotype. We followed a similar approach to the methods described in ref. [18] (see online supplementary material for more details). We calculated the proportions of the population and of cancer cases at different levels of SNP risk and polygenic risk and plotted against each other for comparison purposes. This provides an informative measure of the relationship between risk distribution in the population and among cancer cases. In the hypothetical future when an individual's polygenic risk can be estimated with a high degree of accuracy, either from family history or because most of the currently theoretical polygenotype is accounted for by known variants, these measures could be used to estimate what proportions of the population would need to be monitored/screened/followed in order to detect a particular percentage of OvCs. This could also potentially contribute to stratifying population by OvC risk to enable targeting of effective screening/preventive intervention strategies for appropriate risk groups.

## Results

Data from 1548 OvC cases recruited into the SEARCH study were used for our analyses. Female relatives of probands included 1340 mothers, 1404 sisters and 1144 daughters, of whom 80 were also diagnosed with OvC and 191 with breast cancer. The numbers of probands and their first-degree relatives, the number of OvCs diagnosed in each group and other sample characteristics are summarised in online supplementary table S1. All probands were screened for *BRCA1* and *BRCA2* mutations*,* identifying 44 and 62 carriers, respectively. The loci, minor allele frequencies and ORs of the 17 SNPs used in incorporating their effects into the final model are displayed in online supplementary table S2.

### Segregation analyses for OvC incorporating the effects of *BRCA1* and *BRCA2* mutations

The results for the seven models that incorporate the explicit effects of *BRCA1* and *BRCA2* on OvC risk and that assume cohort-specific incidences are summarised in table 1. All the seven models that accounted for the residual familial aggregation to OvC (in addition to *BRCA1* and *BRCA2)* provided significantly better fit than the model that included only *BRCA1* and *BRCA2* (p <2.8×10^−5^). The worst-fitting model for the residual familial aggregation of OvC, other than *BRCA1* and *BRCA2,* was the major recessive and the most parsimonious was the polygenic model, with AICs of 5772.244 and 5764.372, respectively. Although the mixed models of inheritance all had slightly larger log-likelihoods, they did not improve the fit significantly over the model with only a polygenic component in addition to the *BRCA1* and *BRCA2* effects (LRT p values >0.14). In all models that included a hypothetical third major gene, the relative risk for the susceptible women was very high (ranging between ∼54 and ∼122). The estimated population allele frequency for *BRCA1* and *BRCA2* under the polygenic model were 0.08% (95% CI 0.06% to 0.11%) and 0.26% (95% CI 0.002% to 0.33%), respectively, with a SD of the polygenic component of 1.43 (95% CI 1.1 to 1.86).

**Table 1:** Parameter estimates, goodness-of-fit measures and likelihood ratio tests (LRTs) of the seven cohort-specific models for breast and ovarian cancer

Model	*BRCA1* frequency (95% CI)	*BRCA2* frequency (95% CI)	Major gene frequency (95% CI)	Major gene log relative risk (95% CI)	Polygenic SD (95% CI)	Log-likelihood	AIC	LRT p value
Base	0.00081 (0.00061 to 0.0011)	0.0026 (0.0020 to 0.0033)	–	–	–	−2892.237	5788.474	5.11E-06
Major dominant	0.00079 (0.00060 to 0.0011)	0.0026 (0.0020 to 0.0032)	0.00025 (0.000041 to 0.0015)	4.8 (3.3 to 6.2)	–	−2880.343	5768.686	0.047
Major recessive	0.00080 (0.00060 to 0.0011)	0.0026 (0.0020 to 0.0032)	0.085 (0.017 to 0.33)	4.0 (2.0 to 6.0)	–	−2882.122	5772.244	0.0079
Major general	0.00079 (0.00060 to 0.0011)	0.0026 (0.0020 to 0.0032)	0.00025 (0.00020 to 0.0033)	4.8 (3.3 to 6.3)	–	−2880.335	5770.67	0.013
				7.4 (−14.1 to 28.8)				
Polygenic	0.00079 (0.00060 to 0.0011)	0.0026 (0.0020 to 0.0033)	–	–	1.43 (1.10 to 1.86)	−2879.186	5764.372	0.28
Mixed dominant	0.00079 (0.00059 to 0.0011)	0.0026 (0.0020 to 0.0032)	0.00023 (0.000023 to 0.0022)	4.7 (2.8 to 6.6)	1.09 (0.64 to 1.86)	−2877.289	5764.576	0.91
Mixed recessive	0.00079 (0.00060 to 0.0011)	0.0026 (0.0020 to 0.0032)	0.076 (0.020 to 0.25)	3.7 (1.5 to 5.9)	1.19 (0.74 to 1.91)	−2878.374	5768.806	0.14
Mixed general	0.00079 (0.00059 to 0.0011)	0.0026 (0.0020 to 0.0032)	0.00023 (0.000023 to 0.0023)	4.7 (2.8 to 6.6)	1.09 (0.64 to 1.86)	−2877.283	5766.566	
				9.4 (−20.5 to 39.3)				

AIC, Akaike's information criterion; LRT p value, probability of the difference between log-likelihoods comparing each model against the mixed general model.

### Predicted number of *BRCA1* and *BRCA2* mutation carriers and family members diagnosed with OvC

The expected numbers of *BRCA1* and *BRCA2* mutation carriers computed under each of the models are displayed in table 2. In line with magnitude of the log-likelihoods, all seven models gave similar predictions that were noticeably more accurate than the model that did not allow for additional residual familial aggregation other than the effects of BRCA1 and BRCA2. The polygenic model performed best for predicting the number of *BRCA2* mutation carriers and there was a slight improvement in accuracy of *BRCA1* number under the mixed models. In comparison, under the current implementation of BOADICEA, the predicted *BRCA1* numbers were very close to the observed values but the number of *BRCA2* carriers was substantially underpredicted (p value for difference between observed and expected number of mutations=4.64E-16).

**Table 2:** Number of mutation carriers predicted by each model and comparison with observed numbers

Model for the residual familial aggregation	Observed *BRCA1* carriers	Expected *BRCA1* carriers	Observed *BRCA2* carriers	Expected *BRCA2* carriers	χ^2^ value*
Baseline	44	56.95	62	63.59	2.98
Polygenic	44	49.32	62	61.98	0.57
Dominant major	44	55.62	62	63.08	2.45
Recessive major	44	55.97	62	63.11	2.58
General major	44	55.62	62	63.08	2.45
Dominant mixed	44	48.07	62	61.01	0.36
Recessive mixed	44	49.08	62	61.10	0.54
General mixed	44	48.05	62	61.02	0.36
BOADICEA	44	45.76	62	23.03	66.01

*χ^2^ value, value of χ^2^ goodness-of-fit test.

BOADICEA, Breast and Ovarian Analysis of Disease Incidence and Carrrier Estimation Algorithm.

Similarly, when computing the expected number of families with a mother, a sister or mother and sister diagnosed with OvC, the predicted numbers were closer to that observed for the polygenic and mixed models of inheritance (see online supplementary table S3).

### Predicting future OvC risks

We estimated the probabilities of developing OvC for a 50-year-old woman born in 1940, with the following family histories: (i) no information on relatives; (ii) having a mother and sister cancer free at ages 65 and 50; (iii) mother and sister diagnosed with OvC at ages 65 and 50; and (iv) and (v) with both combinations of one diagnosed and one cancer free at these same ages. We compared these estimates with the risk estimates from the current version of BOADICEA.

**Figure 1:**
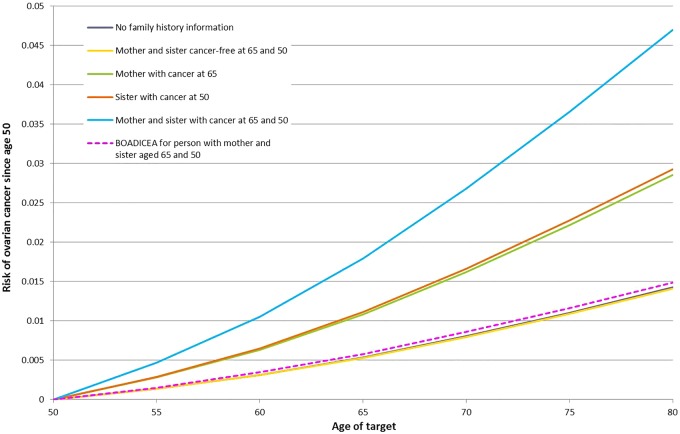
Predicted risks of ovarian cancer over time to a woman born in the 1940 birth cohort without a *BRCA1* or *BRCA2* mutation by family history. The predicted ovarian cancer risks under the most parsimonious model vary by extent of family history of ovarian cancer. In contrast, under the Breast and Ovarian Analysis of Disease Incidence and Carrrier Estimation Algorithm the predicted ovarian cancer risks remain the same under all scenarios.

Figure 1 displays the probabilities of developing OvC for a 50-year old woman without a *BRCA1* or *BRCA2* mutation. Under the best-fitting model, the risk of OvC increases with increasing number of relatives diagnosed with OvC. In contrast, the corresponding predictions under BOADICEA remain the same under all assumptions about family history. Similar patterns are observed when the index female is assumed to carry a *BRCA1*, or a *BRCA2* mutation, where the risks in mutation carriers also depend on the exact family history information (see online supplementary figures S1 and S2). Under BOADICEA, the risks in mutation carriers are not modified by family history and are all very close to the corresponding risks predicted by our polygenic model algorithm for a women with no family history information.

### Incorporating common alleles into the model

The loci, minor allele frequencies and ORs for the 17 SNPs considered are displayed in online supplementary table S2. Under the assumptions that the effects of the SNPs on OvC are all mutually independent and the same for *BRCA1* carriers, *BRCA2* carriers and non-carriers, each observed SNP profile was translated into a PRS. This PRS was assumed to have a centralised normal distribution with a variance of 0.0915, explaining about 4.5% of the total polygenic variance in our model.

**Figure 2:**
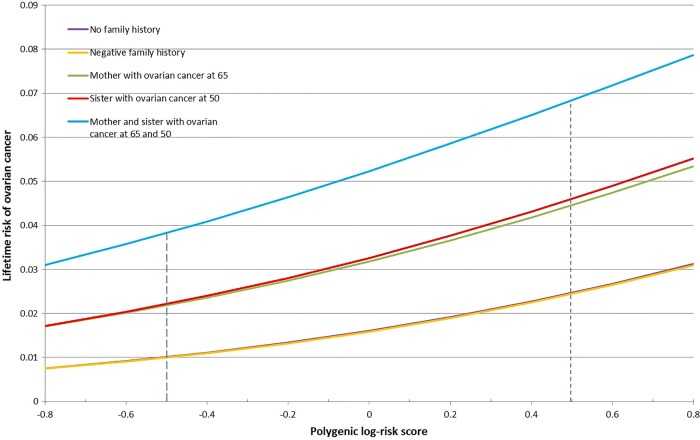
Lifetime risks of ovarian cancer to a 20-year-old born in the 1940 birth cohort without a *BRCA1* or *BRCA2* mutation with different polygenic risk score (PRS) and family history. Graph of the change in probabilities of developing ovarian cancer by age 80 as PRS increases from −0.8 to 0.8, to a 20 year old with five different family histories. The two dotted lines, at −0.496 and 0.496, indicate the PRS of those at the 5th and 95th percentile of risk.

The lifetime risks of OvC to a 20-year-old non*-BRCA1/2* mutation carrier, conditional on known PRS and family history of OvC, are shown in figure 2. As expected, the lifetime risk of developing OvC rose exponentially with increasing PRS. For example, the lifetime risk of OvC for a woman without a BRCA1 or BRCA2 mutation but with two affected first-degree relatives is predicted to be >5% if she is at the top 50% of the PRS distribution.

Examples of age-specific risks for a 50-year-old woman at the 5th and 95th percentiles of the PRS and by different family history assumptions are shown in online supplementary figures S3–S5

### Implications of the polygenic model for OvC prevention

**Figure 3:**
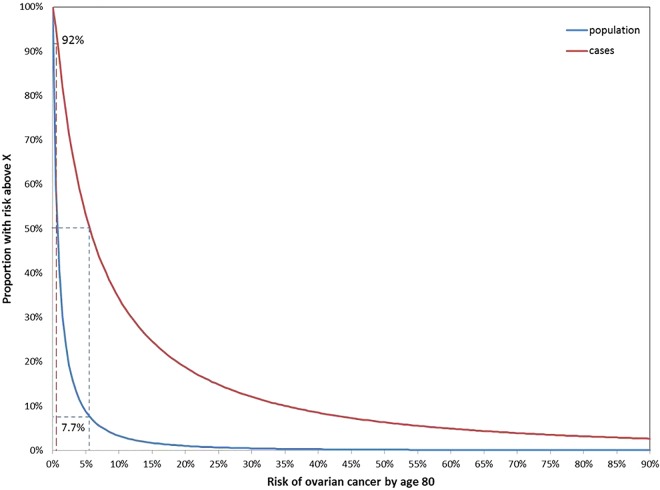
Proportion of population above a specified absolute risk of ovarian cancer and proportion of cases occurring in that fraction of the population. Half the population have an absolute risk of ovarian cancer greater than 0.72% by age 80 and 92% of all cases occur in this half of the population. Half of all cancers occur in the 7.7% of the population with risk higher than 5.6%.

For a polygenic log-risk with the SD of 1.434, estimated under the best-fitting segregation analysis model, and assuming a baseline population OvC risk of 0.02 by age 80, the half of the population at higher risk accounts for 92% of all OvCs. Figure 3 displays the proportion of the population that have a risk greater than a given level and the proportion of the cases predicted to occur within this subgroup. From these curves, it can be seen that 50% of all cancers occur in the 7.7% of the population with a risk of 5.6% or more.

**Figure 4:**
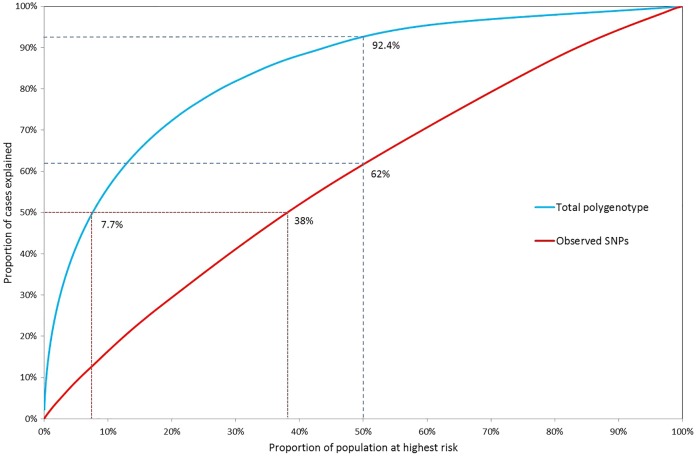
Proportion of cases accounted for by a given proportion of the population above a specified risk of ovarian cancer according to the total polygenic risk and the observed 17 SNP distribution. Under the total polygenic risk distribution, 50% of cancers occur in the 7.7% of the population at highest risk and 92.4% of cancers occur in the half of the population at greater-than-average risk, whereas under the 17 SNP only 62% of cancers occur in the 50% at higher risk and 50% of cases are spread among almost 40% of the population at highest risk.

In figure 4, the population proportions are plotted against the case proportions accounted for, for the polygenic log-risk distributions and the combined SNP-effect distributions. The total known variance of the effects of 17 known SNPs is 
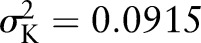
 (see online supplementary material and methods). Due to the low known variance, the distinction between population and case risk is very low for the 17 SNPs alone.

## Discussion

We used complex segregation analysis to develop a risk-prediction model for familial OvC that incorporates the effects of *BRCA1* and *BRCA2* mutations, family history and several newly established common OvC susceptibility alleles using data from a population-based study of OvC cases in the UK. Our model accounts for the familial aggregation of OvC and helps inform a major unresolved clinical question on how to counsel women with family history of OvC but without *BRCA1* or *BRCA2* mutations.

The most parsimonious model included the effects of *BRCA1* and *BRCA2* mutations together with a polygenic component. This suggests that most of the familial aggregation not accounted for by *BRCA1* and *BRCA2* consists of the effects of a large number of genetic variants, each having small contributions to the OvC familial risk. This is in line with results from recent OvC GWAS^19–22^ that have demonstrated that common low-risk OvC susceptibility alleles exist. Parallel studies in breast cancer suggested that thousands of such genetic susceptibility alleles are likely to exist, which explain a substantial fraction of the unexplained genetic variability.^23^ A similar model is likely to apply to OvC. A model that included an additional, dominantly inherited, high-penetrance gene had the highest likelihood. Such a model could reflect the joint effects of other rare OvC susceptibility variants that confer higher risks collectively. However, our analysis may be underpowered as this model did not fit significantly better than the polygenic model.

Previous OvC segregation analyses^24^ ^25^ that accounted for *BRCA1* and *BRCA2* mutations were based on 10-fold smaller sample set of high-risk OvC families and did not investigate polygenic models for the residual familial aggregation of OvC. In contrast to the present study, those studies found no significant evidence of a third high-penetrance gene in addition to *BRCA1* and *BRCA2*. The difference could be explained primarily by the much lower power of those analyses due the smaller sample size but also due to the fact the ascertainment adjustment involved conditioning on all family phenotypes that imposed a much greater penalty in comparison to the present analysis that used families selected only on the OvC status of the index case.

Under the best-fitting model, the *BRCA1* and *BRCA2* mutation frequencies were estimated to be 0.00079 and 0.0026, respectively, corresponding to a carrier frequency of 1 in 630 population for *BRCA1* and 1 in 195 population for *BRCA2.* These were higher than the BOADICEA estimates of 0.0006 for *BRCA1* and 0.001 for *BRCA2,*^10^ but the difference was significant only for *BRCA2* (p values 0.13 and 0.00002). This was also reflected in the significant underprediction of *BRCA2* mutations under the BOADICEA model in the current dataset. This difference between the studies is probably partly due to the data sources and differences in the mutation screening techniques. The 2785 families used to fit the BOADICEA algorithm were ascertained primarily through population-based patients with breast cancer. This source of difference would be in line with the fact that BOADICEA was found to predict *BRCA1* and *BRCA2* mutations and breast cancer risk well in independent datasets of families with breast cancer.^10^ ^26–28^ BOADICEA has not been evaluated so far in families ascertained on the basis of OvC only. Another possible factor is the mutation screening methods. The current study is based on currently available sequencing technologies^6^ that are estimated to be more sensitive in detecting mutations than the techniques used in the late 1990s.^29^ Moreover, the knowledge of which mutations are actually pathogenic has improved substantially over time.^30^ Both of these factors could contribute to higher mutation frequencies, although it is unclear why the difference is only significant for *BRCA2.* An alternative explanation could be a differential response rate for participating in the present study between mutation carriers and non-carriers. *BRCA1* and *BRCA2* mutations have both been associated with improved short-term OvC survival. In particular, *BRCA2* mutation carriers have been reported to have a better prognosis.^31^ ^32^ If women with short prognosis are more likely to participate in the study, this could potentially lead to an overestimation of the mutation frequency. However, data on response differences by prognostic characteristics are not available to assess this.

In the long term, we expect that these differences will be resolved by fitting a single algorithm to all available data that models comprehensively both the genetic susceptibility to breast cancer and OvC. However, at this stage this is not feasible based on current technologies due to computational complexities (in particular, the number of underlying genotypes in the models). The current approach aims to develop separate algorithms for the susceptibility to breast cancer and OvC  that individually incorporate the explicit effects of all observed and unobserved genetic variants such that we obtain accurate risks of each cancer. Validation studies in independent datasets will determine the most appropriate model for use in each context. As technologies evolve over time, in the long term we expect to synthesise the models into a single algorithm.

In our analyses, we took account of OvCs occurring after a breast cancer diagnosis, assuming the OvC incidence remains the same before and after the breast cancer diagnosis. Repeating the analysis but censoring at the first cancer yielded similar results (eg, under the polygenic model *BRCA1* mutation frequency was estimated to be 0.083% and *BRCA2* mutation frequency was 0.27% with a polygenic SD of 1.46). Therefore, our results were not sensitive to these assumptions.

In our analysis, we aimed to include only epithelial OvCs. However, subsequent to the model fitting process, additional pathology information became available, which revealed 41 of the probands’ tumours to be non-epithelial OvCs. This consisted of one *BRCA2* carrier and 40 non-carriers, were non-epithelial OvCs. Refitting the models using only epithelial OvCs had very little effect on results. Under the polygenic model, the estimated *BRCA1* and *BRCA2* mutation frequencies were 0.081% and 0.26%, polygenic SD was 1.44 and the estimated numbers of *BRCA1* and *BRCA2* carriers were 48.6 and 60.8, respectively.

Our models assumed that the mutation testing sensitivities were 0.9 for both *BRCA1* and *BRCA2.* Obtaining exact estimates is difficult, but in practice mutation sensitivities could be lower. We refitted the models using a sensitivity parameter of 0.83 for *BRCA1* and 0.76 for *BRCA2.*^6^ Under the polygenic model, the estimated *BRCA1* and *BRCA2* mutation frequencies were slightly higher at 0.086% and 0.3%, respectively, and the polygenic SD decreased slightly to 1.375, but none of these were significantly different than the results under a sensitivity of 0.9. These patterns are expected as the mutation frequency and mutation screening sensitivity parameters are confounded.

One possible source of bias in our analysis is the possibility of errors in the reporting of family cancer history. However, previous studies have found reported OvC history in first-degree relatives to be reasonably accurate (83.3% probability of agreement between reported OvC status in first-degree relatives and established status).^33^ ^34^ Therefore, the fact that the OvC diagnoses in relatives are not confirmed is unlikely to have a great impact on our results. Another possible weakness of our study is the usage of external estimates of breast cancer and OvC relative risks to *BRCA1* and *BRCA2* mutation carriers. However, due to the small number of mutation carriers in the SEARCH dataset, it was not possible to estimate reliably the cancer risks for BRCA1 and BRCA2 mutation carriers. The estimates used were based on some of the largest studies available.^10^ ^35^ Future studies should aim to analyse all the data jointly.

Under our models, the probabilities of developing OvC increase with the number of OvCs in relatives, while under BOADICEA^10^ the risks remain invariable, at values very close to those we predicted for someone with no recorded family history, which for non-BRCA1 or non-BRCA2 carriers is close to the population risk. This is due to the fact that BOADICEA, along with other previously developed algorithms such as BRCAPRO,^3^ uses only *BRCA1* and *BRCA2* mutations to model genetic susceptibility to OvC. As a result, under BOADICEA and BRCAPRO, OvC risks are determined only by the *BRCA1* and *BRCA2* mutation status, no matter what their family history is. Three quarters of OvC familial relative risk is not accounted for by *BRCA1* and *BRCA2* mutations;^1^ therefore, the present models are more realistic. As it stands, BOADICEA and BRCAPRO could underestimate the risk to many individuals with a family history of OvC but no identified mutations.

In all models incorporating a polygenic component or known SNPs, the effects were assumed to be the same for carriers of a *BRCA1* or *BRCA2* mutation and non-carriers. This assumption is supported by recent studies^17^ ^36^ ^37^ where all but one of the OvC loci identified through GWAS were found to be associated with risk to a similar relative extent in *BRCA1* and *BRCA2* carriers and non-carriers.^38^ If future studies identify additional *BRCA1-*specific or *BRCA2-*specific modifiers of risk, it should be possible to extend the present model to allow for this level of complexity.

Although we have incorporated the explicit effects of the common low-risk alleles, future efforts should focus on incorporating the explicit effects of other intermediate risk OvC susceptibility variants such as *RAD51C, RAD51D* and *BRIP1.*^39–41^ However, prior to incorporating those into risk prediction models, it is critical to obtain precise estimates of the risks conferred by such mutations that currently are not available.

We also used our models to investigate the possible implications for OvC risk stratification in the population. Using the parameters from the polygenic model, we estimate that 50% of OvCs occur within 7.7% of the population at highest risk. Meanwhile, half of the population at lower risk is forecast to contain only 1 in 13 cancer cases. Targeting the 10% at highest polygenic risk for preventative measures or excluding the low-risk half could therefore lead to a much more efficient distribution of resources. However, to achieve this will require that we identify all the genetic factors that contribute to polygenic inheritance. The almost flat curve in figure 4 from the SNP log-risk distribution, with 50% of the population at higher risk predicted to contain around only 60% of cases, suggests very low power to discriminate between high-risk and low-risk individuals on SNP profiles alone. It is perhaps not surprising as currently only 4.5% of the OvC polygenic variance is accounted for by known low-penetrance genetic variants. However, the currently known SNP profiles in combination with family history information and other risk factors for the disease are expected to have a greater impact for individualised OvC risk prediction, as demonstrated by our model.

Our model can be used in the genetic counselling process of women with family history of OvC as well as for counselling women both with and without *BRCA1* or BRCA2 mutations. This would be helpful to both *BRCA1* and *BRCA2* carriers and non-carriers while making decisions regarding clinical interventions following counselling. Probabilities of developing OvC based on family history, *BRCA1, BRCA2* mutation status and/or polygenic risk could be used to assess the risk to an individual and to discriminate between high-risk and low-risk individuals, which may in time prove useful for targeting appropriate interventions.

## Future research

Although the mutation carrier probability algorithms produced very accurate estimates of the number of carriers in the SEARCH data, an external validation is needed to establish the performance of the model in independent datasets and to assess the model performance in predicting OvC risk in prospective studies. Future plans to extend the models include the addition of lifestyle and reproductive factors such as parity, breast feeding and oral contraceptive use,^42^ mutations in genes such as *RAD51C, RAD51D* and *BRIP1* that are known to be associated with OvC risk,^39–41^ competing causes of mortality and differences in the associations of the various risk factors with different OvC morphological subtypes. The ultimate goal is to combine the models within the BOADICEA framework and develop a comprehensive breast and ovarian risk user-friendly prediction tool.

## Supplementary Material

Web supplement
