# Supplementary material for: A risk prediction algorithm for ovarian cancer incorporating BRCA1, BRCA2, common alleles and other familial effects
Source: J Med Genet. 2015 May 29;52(7):465–75. doi: 10.1136/jmedgenet-2015-103077 (PMC4501173; doi:10.1136/jmedgenet-2015-103077)
Supplement: Web supplement [file jmedgenet-2015-103077-s1.pdf]

# Supplementary Material

## A Risk Prediction Algorithm for Ovarian Cancer Incorporating *BRCA1, BRCA2,* Common Alleles and Other Familial Effects

Sarah Jervis[1], Honglin Song[2], Andrew Lee[1], Ed Dicks[2], Patricia Harrington[2], Caroline Baynes, Ranjit Manchanda[4,5],Douglas F. Easton[1,2], Ian Jacobs[3,4], Paul P.D. Pharoah[1,2], Antonis C. Antoniou[1*]

1 Centre for Cancer Genetic Epidemiology, Department of Public and Primary Care, University of Cambridge

2 Centre for Cancer Genetic Epidemiology, Department of Oncology, University of Cambridge

3 Institute of Human Development, Faculty of Medical & Human Sciences, The University of Manchester and Manchester Academic Health Science Centre

4 Institute for Women's Health, University College London

5 Department of Gynaecological Oncology, St Bartholomew's Hospital

*Address for correspondence:

Antonis C Antoniou

Strangeways Research Laboratory

Department of Public Health and Primary Care

Worts Causeway

Cambridge CB1 8RN

UK

## Methods

### Constraining the overall incidences.

The population incidences of ovarian cancer were constrained to agree with national incidence rates for England and Wales. Let $i\ (t)$ denote the population incidence rate at age *t* and denote the value of the polygenotype *P* by $p_r$ to highlight its dependence on *R*, taking value *r*. Then

$$i(t) = \frac{\sum_{g,p_r} \Pr(g, p_r) f_{g,p_r}(t)}{\sum_{g,p_r} \Pr(g, p_r) S_{g,p_r}(t-1)}$$

where $(g, p_r)$ denotes the major-locus genotype *g* and polygenotype $p_r$. $f_{g,p_r}(t)$ and $S_{g,p_r}(t)$ are the probability of developing ovarian cancer at age *t* and the probability of surviving ovarian cancer by age *t*. The major-gene genotype and polygenotype are assumed to be independent so that $\Pr(g, p_r) = \tau_g \phi_{p_r}$, where $\tau_g$ is the probability of major genotype *g* and $\phi_{p_r}$ is the polygenotype probability, given by the binomial distribution. $f_{g,p_r}(t)$ can be rewritten as $\lambda_{g,p_r}(t) S_{g,p_r}(t-1)$. Thus, using the relationship

$$S_{g,p_r}(t-1) = \exp\left[\sum_{u=0}^{t-1} \lambda_{g,p_r}(u)\right] = \exp\left[\sum_{u=0}^{t-1} \lambda_g(u) e^{p_r}\right] = \exp\left[\sum_{u=0}^{t-1} \lambda_g(u)\right]^{e^{p_r}} = S_g(t-1)^{e^{p_r}}$$

$$i(t) = \frac{\sum_{g,p_r} \tau_g \phi_{p_r} \lambda_g(t) e^{p_r} S_g(t-1)^{e^{p_r}}}{\sum_{g,p_r} \tau_g \phi_{p_r} S_g(t-1)^{e^{p_r}}}$$

The above equation is then used to estimate the baseline hazard function $\lambda_0(t)$

**Including *BRCA1* and *BRCA2* ovarian cancer incidence rates**

The external incidence rates of ovarian cancer in the *BRCA1* and *BRCA2* mutation-positive populations, denoted $b_1(t)$ and $b_2(t)$, are constrained by the following equation, similar to that for general population incidences. $\lambda_{b_i}(t)$ and $S_{b_i}(t)$ are the baseline incidence rate and survival probability of a *BRCAi* carrier, "free" of any polygenic effects.

$$b_i(t) = \frac{\sum_{p_r} \Pr(p_r) f(t \mid BRCAi, p_r)}{\sum_{p_r} \Pr(p_r) S(t-1 \mid BRCAi, p_r)} = \frac{\sum_{p_r} \phi_{p_r} f_{b_i}(t \mid p_r)}{\sum_{p_r} \phi_{p_r} S_{b_i}(t-1 \mid p_r)}$$

$$= \frac{\sum_{p_r} \phi_{p_r} S_{b_i}(t-1 \mid p_r) \lambda_{b_i}(t) \exp(p_r)}{\sum_{p_r} \phi_{p_r} S_{b_i}(t-1 \mid p_r)} = \frac{\lambda_{b_i}(t) \sum_{p_r} \phi_{p_r} S_{b_i}(t-1)^{e^{p_r}} \exp(p_r)}{\sum_{p_r} \phi_{p_r} S_{b_i}(t-1)^{e^{p_r}}}$$

Solving this equation for the baseline incidence gives us

$$\lambda_{b_i}(t) = \frac{b_i(t) \sum_{p_r} \phi_{p_r} S_{b_i}(t-1)^{e^{p_r}}}{\sum_{p_r} \phi_{p_r} S_{b_i}(t-1)^{e^{p_r}} e^{p_r}},$$

This can be solved iteratively, starting at $S_{b_i}(0) = 1$ and updating the baseline hazard and survival functions alternatively.

The population incidence rate equation becomes:

$$i(t) = \frac{\sum_{g,p_r} \Pr(g, p_r) f_{g,p_r}(t)}{\sum_{g,p_r} \Pr(g, p_r) S_{g,p_r}(t-1)} = \frac{\sum_{g,p_r} \Pr(g, p_r) f_{g,p_r}(t)}{\sum_{p_r} \phi_{p_r} \left( \sum_{k=0}^{K-1} \tau_k S_k(t-1)^{e^{p_r}} \right)}$$ ,where k represents the major genotypes (0=non-carrier, 1=BRCA1 carrier, 2=BRCA2 carrier, 3,..K-1= carriers of

other hypothetical ovarian cancer susceptibility genetic variants). Multiplying both sides by the quotient gives:

$$i(t)\sum_{p_r}\phi_{p_r}\left(\sum_{k=0}^{K-1}\tau_k S_k(t-1)^{e^{p_r}}\right)=\sum_{g,p_r}\Pr(g,p_r)f_{g,p_r}(t)$$
$$=\sum_{p_r}\phi_{p_r}\left(\lambda_0\tau_0 S_0(t-1)^{e^{p_r}}+\sum_{k=3}^{K-1}\lambda_0\tau_k S_k(t-1)^{e^{p_r}}r(k)\right)e^{p_r}+\sum_{p_r}\phi_{p_r}\left(\tau_1 f_{1,p_r}(t)+\tau_2 f_{2,p_r}(t)\right),$$

Rearrangement of this and use of parts of the *BRCA1* and *BRCA2* incidence rate equations produce the following equation for the baseline hazard function.

$$\lambda_0(t)=\frac{i(t)\sum_{p_r}\phi_{p_r}\left(\sum_{k=0}^{K-1}\tau_k S_k(t-1)^{e^{p_r}}\right)-\sum_{p_r}\phi_{p_r}\left(\tau_1 f_{1,p_r}(t)+\tau_2 f_{2,p_r}(t)\right)}{\sum_{p_r}\phi_{p_r}\left(\tau_0 S_0(t-1)^{e^{p_r}}+\sum_{k=3}^{K-1}\tau_k S_k(t-1)^{e^{p_r}}r(k)\right)e^{p_r}}$$
$$=\frac{i(t)\sum_{p_r}\phi_{p_r}\left(\sum_{k=0}^{K-1}\tau_k S_k(t-1)^{e^{p_r}}\right)-\sum_{p_r}\phi_{p_r}\left(\tau_1\lambda_{b_1}(t)S_{b_1}(t-1)^{e^{p_r}}+\tau_2\lambda_{b_2}(t)S_{b_2}(t-1)^{e^{p_r}}\right)\exp(p_r)}{\sum_{p_r}\phi_{p_r}\left(\tau_0 S_0(t-1)^{e^{p_r}}+\sum_{k=3}^{K-1}\tau_k S_k(t-1)^{e^{p_r}}r(k)\right)\exp(p_r)}$$

which can be solved as part of a multi-step iterative process along with the baseline *BRCA1* and *BRCA2* hazard functions and the survival functions for each major genotype.

For the major gene and mixed models of inheritance the major gene component was based on three genes*: BRCA1*, *BRCA2,* and a third hypothetical gene. Because the probability of having both a *BRCA1* and a *BRCA2* mutation is very small, we coded *BRCA1* and *BRCA2* as a single locus with three alleles: *BRCA1* positive, *BRCA2* positive, and a normal allele. The third gene was diallelic with a normal and a mutant allele, and was assumed to be unlinked to *BRCA1* and *BRCA2*. For simplicity, *BRCA1* mutations were assumed to be dominant over all other alleles and *BRCA2* mutations

were assumed to be dominant over hypothetical third locus disease alleles. There were, therefore, five potential risk categories based on the major genotype: *BRCA1* carriers, *BRCA2* carriers, heterozygotes for the third locus risk allele, homozygotes for the third locus allele and non-carriers.

**Incorporating breast cancer into the model**

Like ovarian cancer, the baseline hazard functions and survival functions are obtained from constraining the national incidence rates.

Under the assumption that breast cancer incidence is independent of the polygenotype in these models, the baseline hazard functions $\lambda_1^b(t)$ and $\lambda_2^b(t)$, for the *BRCA1* and *BRCA2*-mutation-positive populations respectively, are just the incidence rates $inc_1^b(t)$ and $inc_2^b(t)$.

The breast cancer incidence rates for the general population are constrained by the equation:

$$i^b(t) = \frac{\sum_{g,p_r} \Pr(g,p_r) f_g^b(t) S_{g,p_r}^o(t-1)}{\sum_{g,p_r} \Pr(g,p_r) S_{g,p_r}^o(t-1) S_g^b(t-1)} = \frac{\sum_g \tau_g f_g^b(t) \sum_{p_r} \phi_{p_r} S_{g,p_r}^o(t-1)}{\sum_g \tau_g S_g^b(t-1) \sum_{p_r} \phi_{p_r} S_{g,p_r}^o(t-1)},$$

Multiplying by the right-hand quotient and utilising information on the ovarian cancer survival functions gives the equation

$$\begin{aligned} i^b(t) \sum_g \tau_g S_g^b(t-1) \sum_{p_r} \phi_{p_r} S_g^o(t-1)^{e^{p_r}} &= \sum_g \tau_g S_g^b(t-1) \lambda_g^b(t) \sum_{p_r} \phi_{p_r} S_g^o(t-1)^{e^{p_r}} \\ = \lambda_0^b(t) \left( \tau_0 S_0^b(t-1) \sum_{p_r} \phi_{p_r} S_0^o(t-1)^{e^{p_r}} + \sum_{k=3} \tau_k S_k^b(t-1) \sum_{p_r} \phi_{p_r} S_k^o(t-1)^{e^{p_r}} \right) &, \\ + \lambda_1^b(t) \tau_1 S_1^b(t-1) \sum_{p_r} \phi_{p_r} S_1^o(t-1)^{e^{p_r}} + \lambda_2^b(t) \tau_2 S_2^b(t-1) \sum_{p_r} \phi_{p_r} S_2^o(t-1)^{e^{p_r}} & \end{aligned}$$

Rearranging this gives the following equation for the baseline hazard function:

$$\lambda_0^b(t) = \frac{i^b(t)\sum_{k=0}\tau_k S_k^b(t-1)\sum_{p_r}\phi_{p_r} S_k^o(t-1)^{e^{p_r}} - \lambda_1^b(t)\tau_1 S_1^b(t-1)\sum_{p_r}\phi_{p_r} S_1^o(t-1)^{e^{p_r}} - \lambda_2^b(t)\tau_2 S_2^b(t-1)\sum_{p_r}\phi_{p_r} S_2^o(t-1)^{e^{p_r}}}{\tau_0 S_0^b(t-1)\sum_{p_r}\phi_{p_r} S_0^o(t-1)^{e^{p_r}} + \sum_{k=3}\tau_k S_k^b(t-1)\sum_{p_r}\phi_{p_r} S_k^o(t-1)^{e^{p_r}}}$$

**Incorporating SNPs into the risk prediction algorithm**

Given that the risk $P(y_1^* \mid \underline{y})$ of an individual developing ovarian cancer between ages $t_0$ and $t_1$ is equal to $\frac{\sum_{t=t_0}^{t=t_1} P(\underline{y}_t^*)}{P(\underline{y})}$ where $P(\underline{y}_t^*)$ is the probability of the family phenotypes including the individual developing OvC at time point $t$, the probability $P(y_1^* \mid \underline{y}, P_{Ki})$, of the same event conditional on the family genotype and the observed SNP genotypes, is equal to $\frac{\sum_{t=t_0}^{t=t_1} P(\underline{y}_t^*, P_{Ki})}{P(\underline{y}, P_{Ki})} = \frac{\sum_{t=t_0}^{t=t_1} P(\underline{y}_t^* \mid P_{Ki}) P(P_{Ki})}{P(\underline{y} \mid P_{Ki}) P(P_{Ki})}$.

This can then be rewritten in terms of the $n$th total polygenotype of the proband as

$$\frac{\sum_{t=t_0}^{t=t_1} P(\underline{y}_t^*, P_{Ki})}{P(\underline{y}, P_{Ki})} = \frac{\sum_{t=t_0}^{t=t_1} \sum_{n=0}^{2N+1} P(\underline{y}_t^* \mid P_{Ki}) P(P_{Ki} \mid P_i^n) P(P_i^n)}{\sum_{n=0}^{2N+1} P(\underline{y} \mid P_{Ki}) P(P_{Ki} \mid P_i^n) P(P_i^n)} = \frac{\sum_{t=t_0}^{t=t_1} \sum_{n=0}^{2N+1} P(\underline{y}_t^*, P_i^n) P(P_{Ki} \mid P_i^n)}{\sum_{n=0}^{2N+1} P(\underline{y}, P_i^n) P(P_{Ki} \mid P_i^n)},$$

where $P(P_{Ki} \mid P_i^n)$ is the conditional normal density function given by

$$P_{Ki} \mid P_i^n \sim N\left( P_i^n \frac{\sigma_K^2}{\sigma_K^2 + \sigma_U^2}, \frac{\sigma_U^2 \sigma_K^2}{\sigma_K^2 + \sigma_U^2} \right)$$

$P(y_1^* \mid \underline{y})$ can also be written in terms of $P_i$ as

$$P(y_1^* \mid \underline{y}) = \frac{\sum_{t=t_0}^{t=t_1} P(\underline{y}_t^*)}{P(\underline{y})} = \frac{\sum_{t=t_0}^{t=t_1} \sum_{n=0}^{2N+1} P(\underline{y}_t^*, P_i^n)}{\sum_{n=0}^{2N+1} P(\underline{y}, P_i^n)},$$

Thus the probability of an individual developing OvC conditional on their observed SNP genotype is the ratio of two likelihood function sums, each term of which can be

computed as the corresponding term in the family history-conditional risk probability multiplied by a conditional normal density.

**Distribution of ovarian cancer risk and implications for ovarian cancer prevention.**

Based on our model, given a log-normal polygenic risk of $e^{y_p}$ in the general population, where $y_p$ is predicted to follow a normal distribution with standard deviation $\sigma$ and mean $-\sigma^2/2$, rescaled so that the average risk $E(e^{y_p})$ is equal to 1, it is easily established that the distribution of initial risk among individuals diagnosed with cancer is also log-normal with the log-risk $y_c \sim N(\sigma^2/2, \sigma^2)$ (see [1], methods). Computing the area under the two normal curves to the right of any given risk point gives us an estimate of the proportion of the population with risk greater than a given level and of the proportion of all cancer cases which will occur within this subgroup. Comparing these values gives a potentially informative measure of the relationship between risk distribution in the population and among cancer cases.

A measure of the predictive power of a 17-SNP genotype risk score could be informative as an indicator of how useful these SNPS are in combination for predicting OvC risk-distribution in the general population – a risk score that can be used to identify a high proportion of all cancers in a relatively low proportion of the population is very useful while one which would need almost half the population to be closely monitored to detect little more than 50% of cancers is almost as costly and ineffective as following 50% of the population at random. A comparison of the predictive power of the SNP risk with that of a total polygenic risk based on explicit family history could also give an indication of how much familial OvC still remains unaccounted for.

The combined log-effects of the seventeen SNPs were assumed to have a normal distribution with variance $V = \sum V_i$ where $V_i = \log\left\langle \left( \frac{1 - p_i + p_i \exp(2r_i)}{(1 - p_i + p_i \exp(r_i))^2} \right)^2 \right\rangle$ is the variance of the log-risk distribution from the $i^{th}$ SNP, with frequency $p_i$ and log-risk $r_i$ [2] [3]. The proportions of the population and of cancer cases at different levels of SNP risk and polygenic risk were plotted against each other for comparison purposes.

## Results: Sample statistics, pathology and genetic information.

Supplementary Table 1. Sample size, age and case distribution for the probands and their relatives

| | Probands | | Mothers | | Sisters | | Daughters | |
|---|---|---|---|---|---|---|---|---|
| | <=50 | >50 | Proband<=50 | Proband>50 | Proband<=50 | Proband>50 | Proband<=50 | Proband>50 |
| Individuals | 415 | 1133 | 356 | 984 | 399 | 1005 | 245 | 899 |
| | 1548 | | 1340 | | 1404 | | 1144 | |
| Families (no. with 1 or more) | 415 | 1133 | 356 | 984 | 236 | 588 | 178 | 611 |
| | 1548 | | 1340 | | 824 | | 789 | |
| Mean age (SD) | 43.9 (6.4) | 60.4 (5.7) | 69.9 (10.8) | 74.5 (11.7) | 47.1 (10.4) | 61.2 (11.7) | 20.7 (8.1) | 36.5 (7.2) |
| | 55.9 (9.4) | | 73.3 (11.7) | | 57.2 (13.0) | | 33.1 (9.8) | |
| No. ovarian cancers | 415 | 1133 | 20 | 31 | 5 | 21 | 0 | 3 |
| | 1548 | | 51 | | 26 | | 3 | |

Supplementary table 2: SNPs and associated Odds Ratio estimates used in the construction of the Polygenic Risk Score.

| Locus | SNP | Minor allele frequency | Per-allele odds ratio |
|---|---|---|---|
| 3p157 | rs15789171 | 0.049 | 1.45 |
| 9p16 | rs3814113 | 0.32 | 0.83 |
| 8p129 | rs1400482 | 0.13 | 0.85 |
| 19p17 | rs4808075 | 0.30 | 1.12 |
| 17p40 | rs62065444 | 0.18 | 1.15 |
| 17p43 | rs10069690 | 0.26 | 1.09 |
| 5p1 | rs2252894 | 0.66 | 0.89 |
| 2p176 | rs12450786 | 0.26 | 1.13 |
| 8p82 | rs74544416 | 0.067 | 1.20 |
| 17p33 | rs3744763 | 0.59 | 0.94 |
| 10p22 | rs12779865 | 0.34 | 1.09 |
| 1p36 | rs56318008 | 0.15 | 1.11 |
| 1p34.3 | rs58722170 | 0.23 | 1.08 |
| 4q26 | rs17329882 | 0.24 | 1.09 |
| 6p22.1 | rs1161331104 | 0.31 | 0.93 |
| 9q34.2 | rs635634 | 0.19 | 1.11 |
| 17q11.2 | chr17:29181220:I5 | 0.28 | 0.91 |

| Supplementary Table 3: Predicted number of families with ovarian cancer under each model fitted | | | |
|---|---|---|---|
| | **Families with*:** | | |
| | **Only mother diagnosed with Ovarian cancer** | **Only 1 sister diagnosed with ovarian cancer** | **Mother and 1 sister diagnosed with ovarian cancer** |
| Observed number of families | 38 | 18 | 3 |
| **Model** | | | |
| Base | 19.9 | 7.98 | 0.39 |
| Major Dominant | 29.83 | 10.73 | 2.20 |
| Major Recessive | 26.76 | 18.90 | 1.24 |
| Major General | 29.87 | 10.74 | 2.20 |
| Polygenic | 36.27 | 14.54 | 1.35 |
| Mixed Dominant | 35.11 | 13.50 | 1.95 |
| Mixed Recessive | 33.94 | 17.86 | 1.55 |
| Mixed General | 35.61 | 13.64 | 2.04 |
| *These assume no other cancers, breast or ovarian cancer in other family members (ie the three scenarios are mutually exclusive) | | | |

## Supplementary figures

Supplementary Figure 1: Predicted risks of ovarian cancer over time to a *BRCA1*-carrier born in the 1940 birth cohort by family history.

Supplementary Figure 2: Predicted risks of ovarian cancer over time to a *BRCA2*-carrier born in the 1940 birth cohort by family history.

Supplementary Figure 3: Estimated ovarian cancer cumulative risk to a 50-year old female born in the 1940birth cohort in the general population (family history information not considered), by PRS percentile.

Supplementary Figure 4: Estimated ovarian cancer cumulative risk to a 50-year old female born in the 1940birth cohort with a mother diagnosed with ovarian cancer at 65, by PRS percentile.

Supplementary Figure 5: Estimated ovarian cancer cumulative risk to a 50-year old female born in the 1940birth cohort with mother and sister diagnosed with ovarian cancer at 65 and 50, by PRS percentile.

**Supplementary Figure 1**

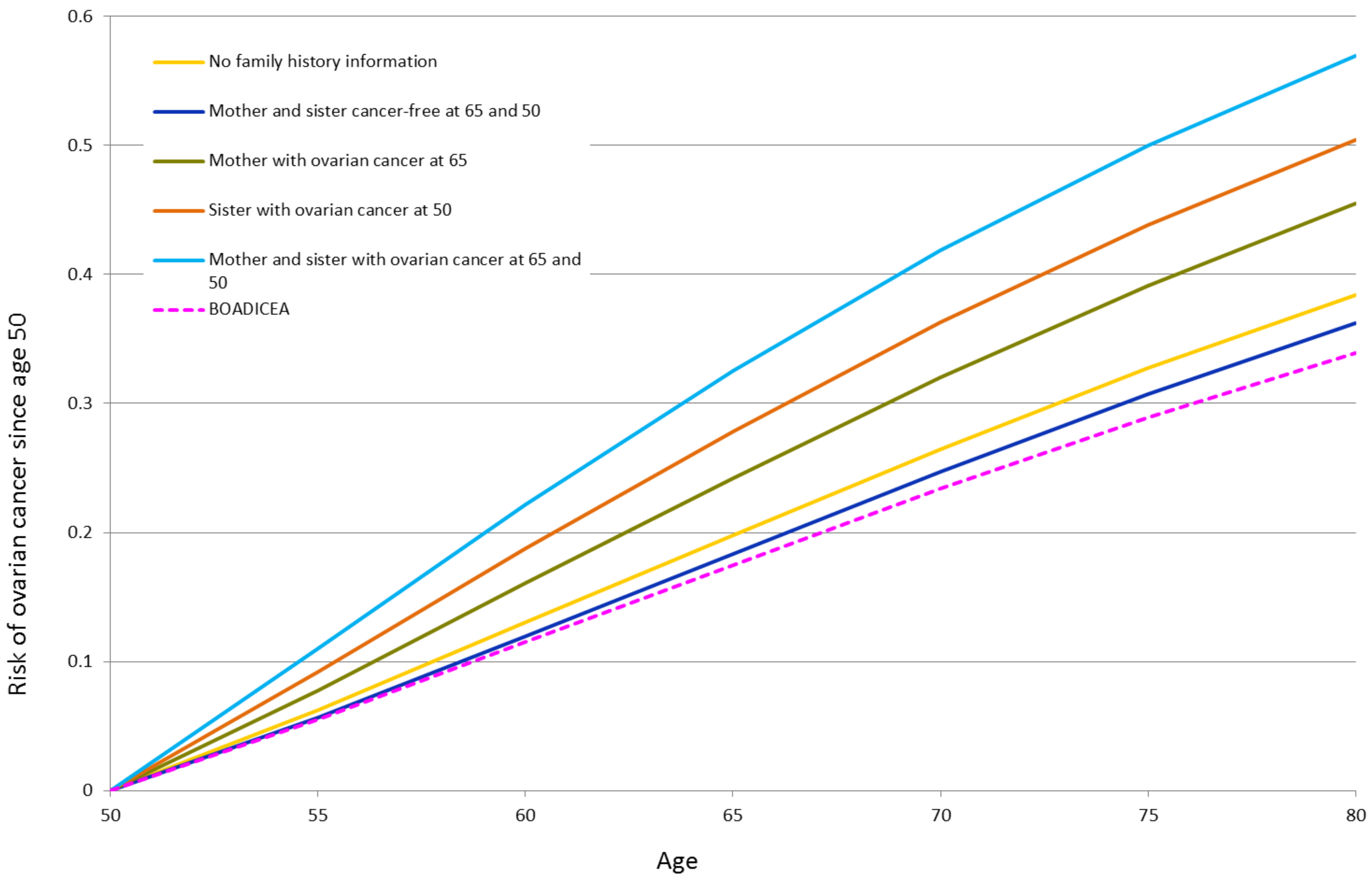

**Supplementary Figure 2**

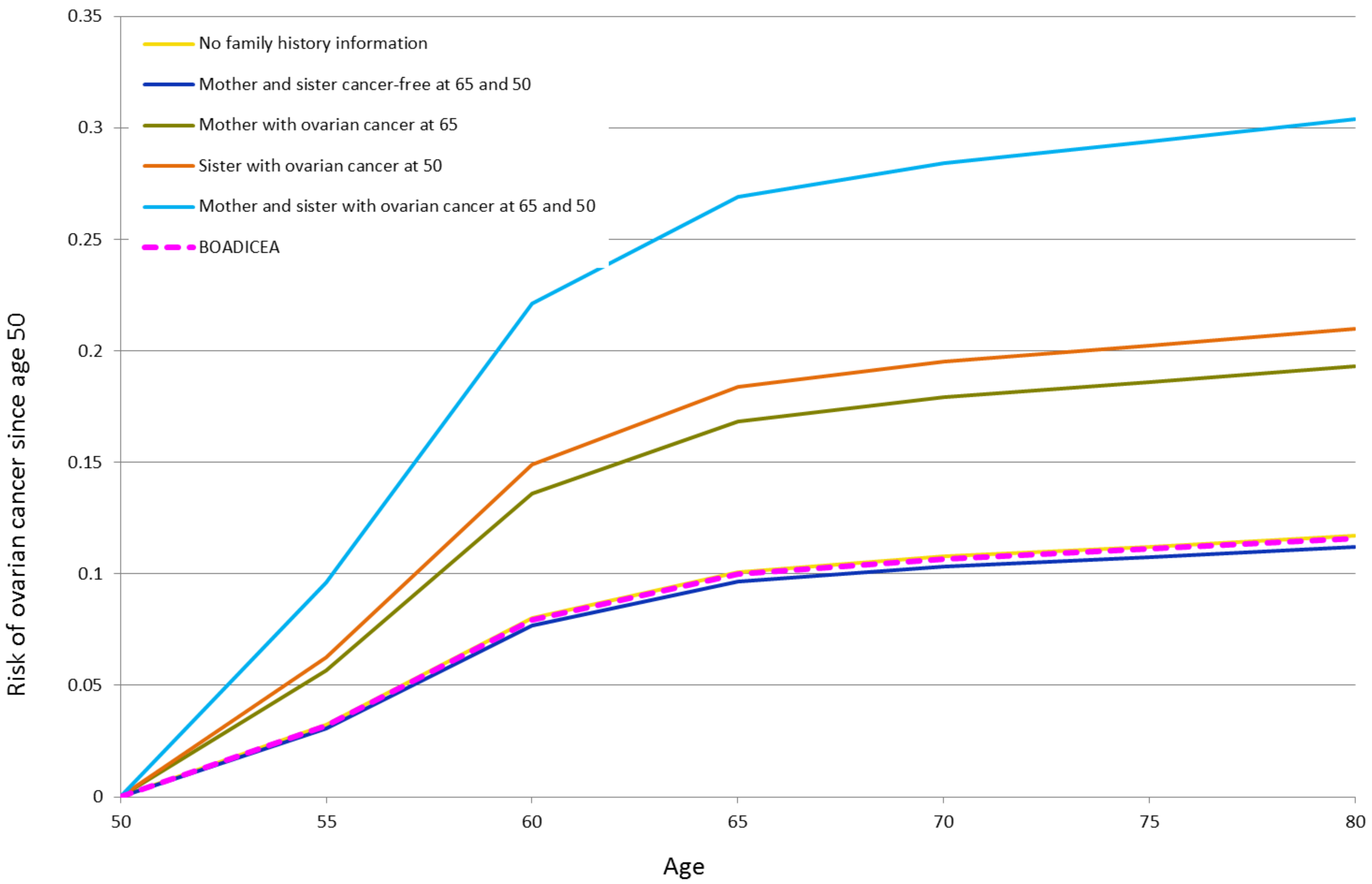

**Supplementary Figure 3**

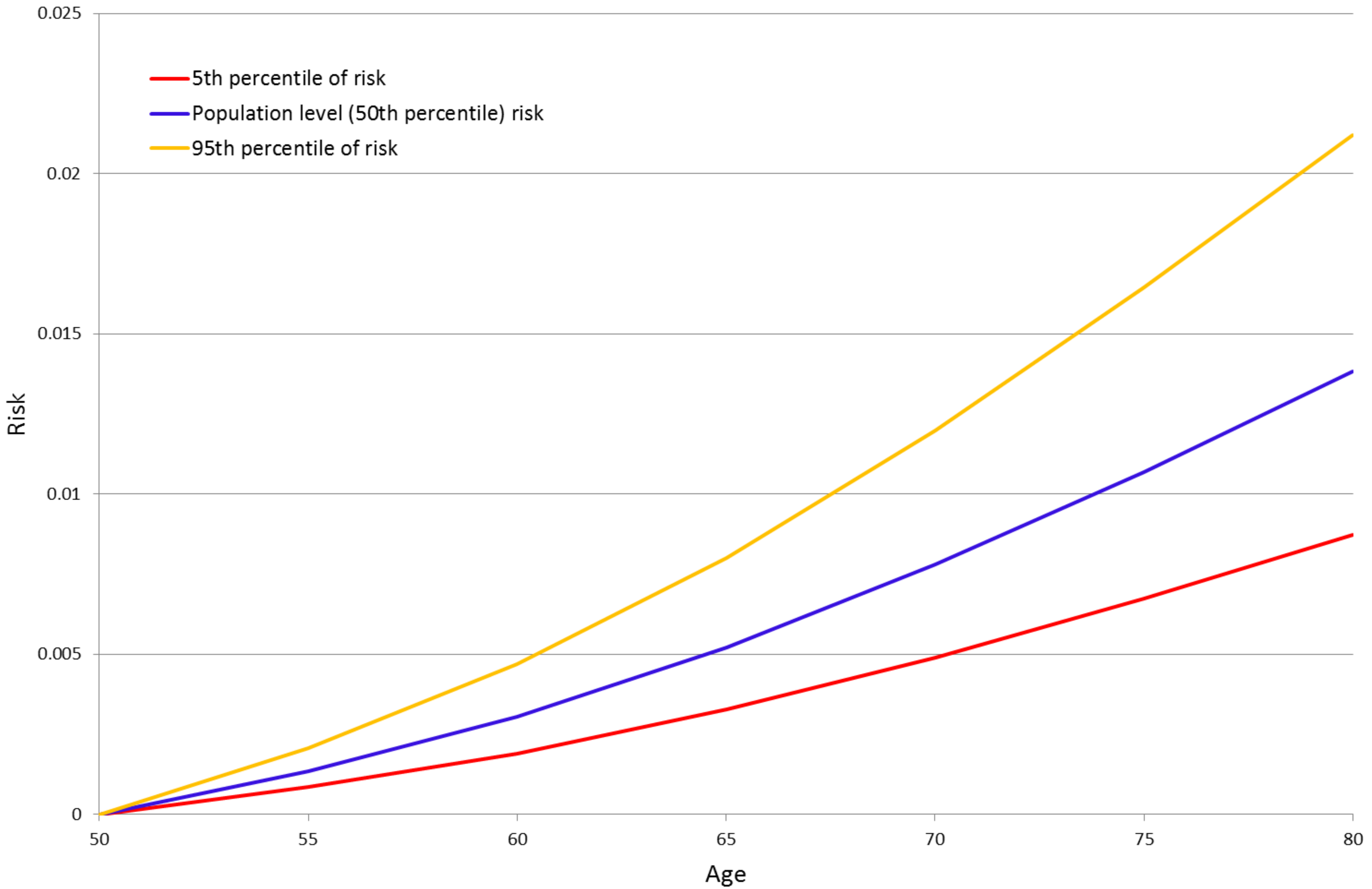

**Supplementary Figure 4**

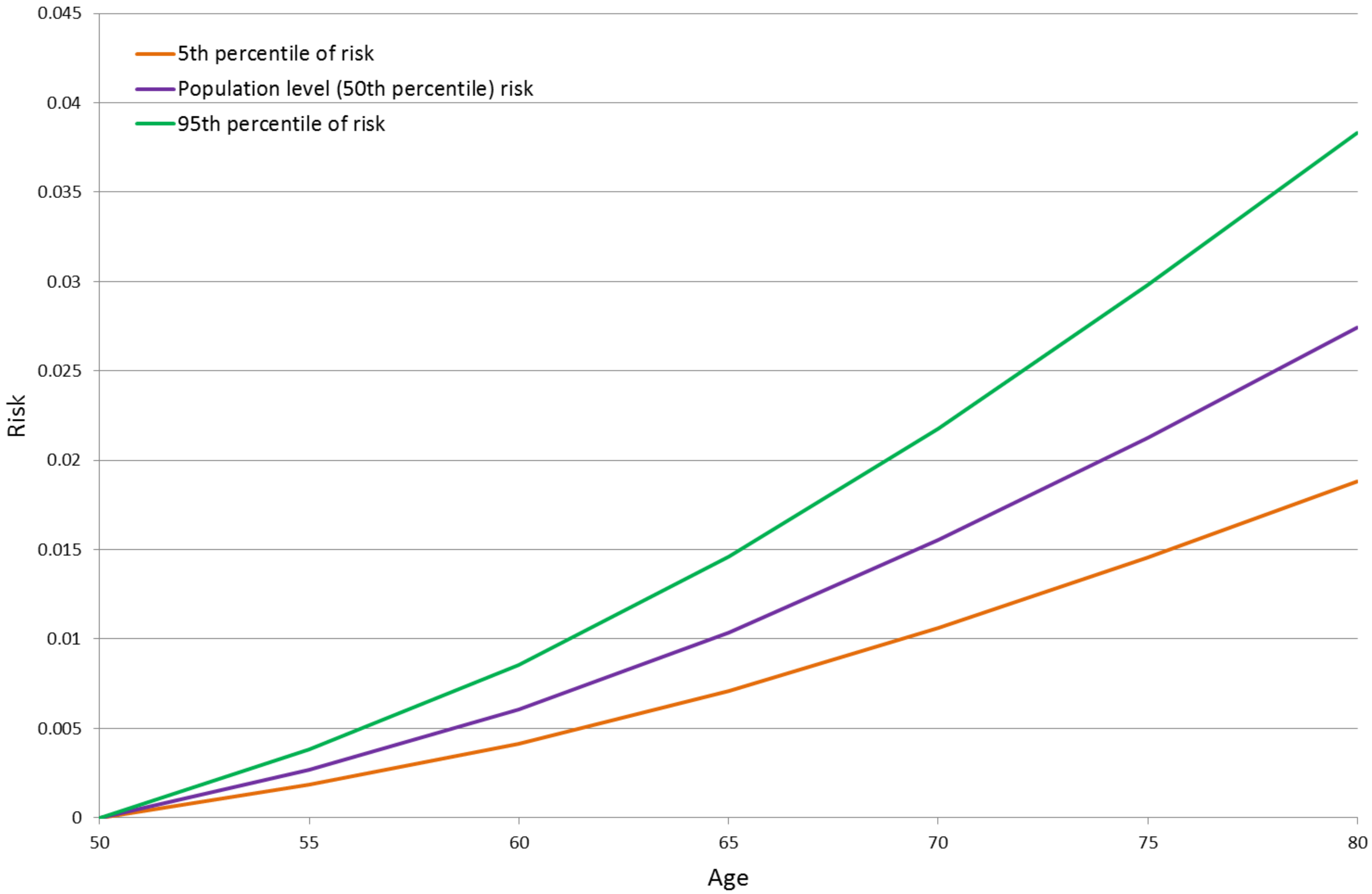

**Supplementary Figure 5**

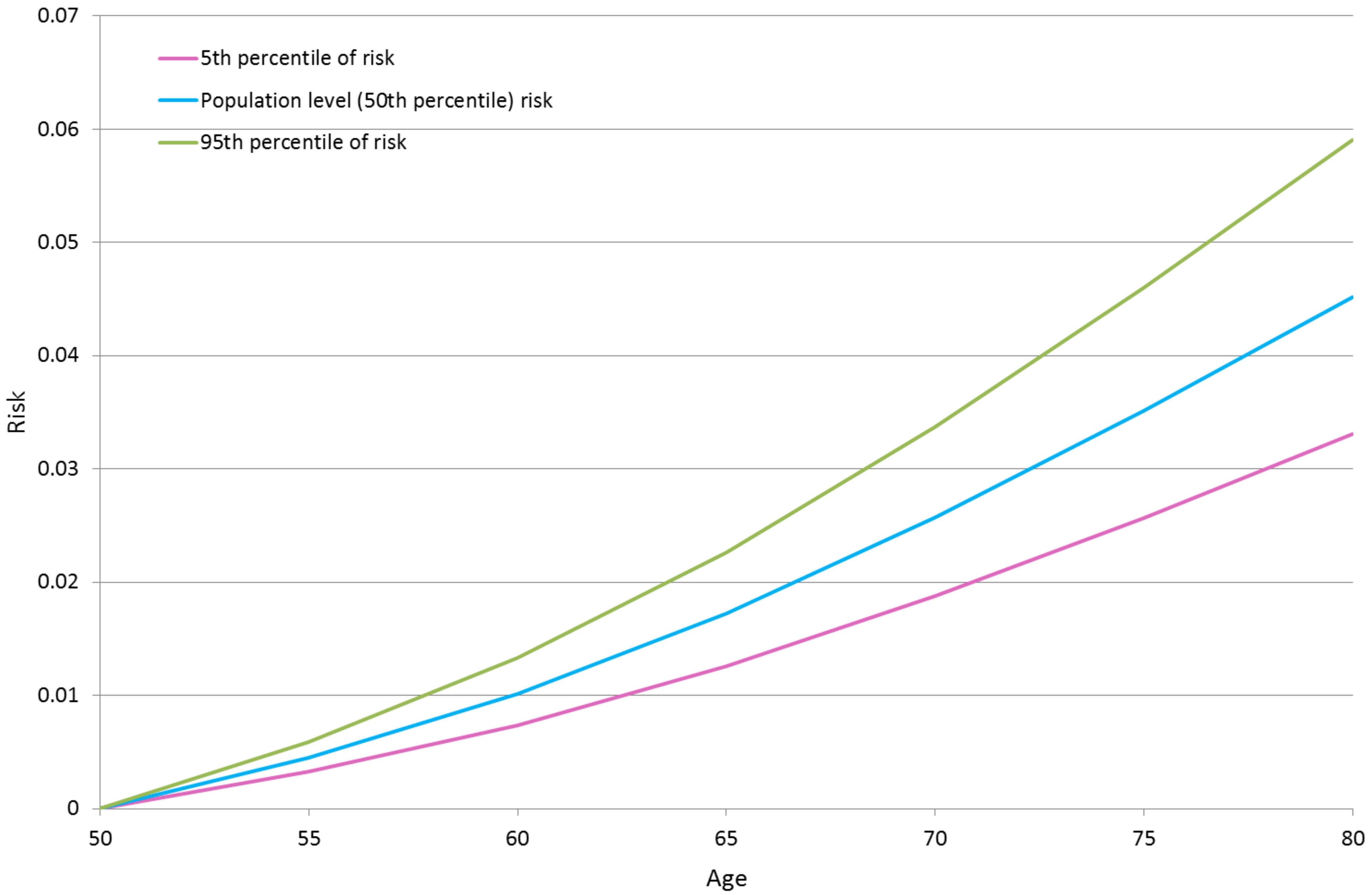

References

1. Pharoah PD, Antoniou A, Bobrow M, Zimmern RL, Easton DF, Ponder BA. Polygenic susceptibility to breast cancer and implications for prevention. Nat Genet. 2002;31(1):33-6.

2. Macinnis RJ, Antoniou AC, Eeles RA, Severi G, Al Olama AA, McGuffog L, Kote-Jarai Z, Guy M, O'Brien LT, Hall AL, Wilkinson RA, Sawyer E, Ardern-Jones AT, Dearnaley DP, Horwich A, Khoo VS, Parker CC, Huddart RA, Van As N, McCredie MR, English DR, Giles GG, Hopper JL, Easton DF. A risk prediction algorithm based on family history and common genetic variants: application to prostate cancer with potential clinical impact. Genet Epidemiol. 2011;35(6):549-56.

3. Antoniou AC, Easton DF. Polygenic inheritance of breast cancer: Implications for design of association studies. Genet Epidemiol. 2003;25(3):190-202.
